# Association of partial adherence (PA) to antiretroviral therapy with hospitalizations and healthcare costs in an HIV population

**DOI:** 10.7448/IAS.15.6.18060

**Published:** 2012-11-11

**Authors:** C Cohen, K Davis, J Meyers

**Affiliations:** 1 Community Research Initiative of New England, Boston, USA; 2 RTI Health Solutions, Health Economics, 200 Park Offices Drive, USA

## Abstract

**Purpose:**

One intrinsic feature of once daily single tablet regimens (STR) in HIV treatment is to prevent missing part of a multi-drug regimen (i.e., partial adherence [PA]). We explored the frequency of PA among HIV patients (pts) treated with multi-pill protease inhibitor (PI), raltegravir (RAL), and non-nucleoside reverse transcriptase inhibitor (NNRTI) regimens and associations with hospitalizations and costs.

**Methods:**

We analyzed healthcare claims from a US Medicaid population. Patients with an HIV diagnosis from 1/09 to 12/11 receiving complete ART (2 NRTIs plus a NNRTI, PI, or RAL) for ≥90 days as a STR or 2+ tablets daily were selected. Adherence and costs were observed from initiation until discontinuation of the entire regimen, switching third component classes, or database end. Adherence was reported as the percent of days (from pharmacy refill data) having a complete regimen, PA, or no medication. A logistic model assessed predictors of hospitalizations and a generalized linear model assessed healthcare costs with covariates for PA and complete non-adherence (CNA), third component, demographics, and prior ART use.

**Results:**

N=1,878 STR, 729 RAL, 3,556 PI, and 775 NNRTI pts. CNA was similar across cohorts: 14.3% (46.4 days[d]) for STR, 14.0% (40.8 d) for RAL, 15.5% (53.0 d) for PI, and 13.5% (51.7 d) for multi pill NNRTI regimens. PA was seen in 12.1% (39.7 d) for RAL, 6.9% (31.7 d) for NNRTI, and 6.6% (26.7 d) for PI regimens. Thus, pts on STR had the highest percent of days (85.7%) with complete adherence to their regimen, vs. pts on multi pill RAL (73.9%), PI (77.9%), and NNRTI (79.6%) regimens. Likelihood of hospitalization significantly increased with both PA and CNA. Compared to <10 days of PA, the risk of hospitalization significantly increased (all p<0.05) with degree of incomplete adherence. For 20–30 days, the odds ratio [OR] was 1.34 for PA, and 1.45 for CNA. The OR was 1.74 for PA and 2.00 for >50 days CNA. Adjusted monthly healthcare costs were significantly greater for patients who were partially adherent for >5% of days (mean [SD] $5,108 [$1,315]) vs. ≤5% of days ($3,602[$894]).

**Conclusions:**

Patients on multi pill PI, RAL, and NNRTI regimens had significantly lower complete adherence vs. STR users, mainly due to PA to a multi-pill regimens. PA and CNA to multi-pill PI, RAL, and NNRTI regimens are independently associated with a significant increase in hospitalizations and healthcare cost.

